# Retraction: Downregulation of OIP5-AS1 affects proNGF-induced pancreatic cancer metastasis by inhibiting p75NTR levels

**DOI:** 10.18632/aging.204491

**Published:** 2023-03-14

**Authors:** Ang Li, Lei Feng, Xiaoya Niu, Qihui Zeng, Bei Li, Zhen You

**Affiliations:** 1 Department of Pancreatic Surgery, West China Hospital of Sichuan University, Chengdu 610041, China; 2 Department of Biliary Surgery, West China Hospital of Sichuan University, Chengdu 610041, China

**Keywords:** pancreatic cancer, long non-coding RNA OIP5-AS1, microRNA-186-5p, nerve growth factor receptor, pro-nerve growth factor

**This article has been retracted:** Aging has completed its investigation of this paper. We found overlap between some of the transwell assay images used in **Figures 5F, 6F, 6H, 7G** and data published by other authors at earlier date [1]. We also found that images of wound healing assay presented in **Figure 7D, F** duplicate images published in unrelated papers [2,3], and identical images of Western blots in **Figure 6K** are presented in two unrelated papers [2,4]. The authors informed Aging that all these issues cannot be resolved and agreed to retract the article.
